# The river and the sea: fieldwork in human ecology and ethnobiology

**DOI:** 10.1186/1746-4269-10-70

**Published:** 2014-10-02

**Authors:** Alpina Begossi

**Affiliations:** UNICAMP (UNICAMP: CAPESCA/NEPA), Campinas, 13083-852 SP Brazil; Fisheries and Food Institute (FIFO), and ECOMAR/UNISANTA, Santos, 11045-040 SP Brazil

## Abstract

This article is a commentary on the experiences that motivated my decision to become a human ecologist and ethnobiologist. These experiences include the pleasure of studying and of having the sense of being within nature, as well as the curiosity towards understanding the world and minds of local people. In particular, such understanding could be driven by addressing the challenging questions that originate in the interactions of such individuals with their natural surroundings. I have been particularly interested in the sea and the riverine forests that are inhabited by coastal or riverine small-scale fishers. Sharing the distinctive world of these fishers enjoyably incited my curiosity and challenged me to understand why fishers and their families ‘do as they do’ for their livelihoods including their beliefs. This challenge involved understanding the rationality (or the arguments or views) that underlies the decisions these individuals make in their interaction with nature. This curiosity was fundamental to my career choice, as were a number of reading interests. These reading interests included political economy and philosophy; evolution and sociobiology; evolutionary, human, and cultural ecology; cultural transmission; fisheries; local knowledge; ecological economics; and, naturally, ethnobiology.

## Early experiences: the pre-human ecology and ethnobiology period

The ability to travel and understand ideas in the real world, including the interface of theory and practice, in addition to my interest in nature and human beings motivated me to study biology and ecology. My interest was powerful. As if a dynamo were inside me, it compelled me to travel throughout Brazil as a way to learn about biological and cultural diversity. Initially, I was attracted to Brazil’s extensive coastline, and then to the country’s immense rivers and forests, particularly Amazonia. In 1977, which was my first year of study toward a Bachelor’s degree in biology at the Federal University of Rio de Janeiro, ten colleagues and I travelled the Brazilian coast from Rio de Janeiro to the Northeast. We stopped in villages and cities on the coasts of Espirito Santo, Bahia, Sergipe, Alagoas, and Pernambuco and continued to Paraiba.

After this first trip to the coast, during my second year, three companions and I journeyed to the Araguaia River in Brazil. The heavy rains and flooding made this trip an adventure, and we eventually arrived at the river. After days of travel by automobile over often unpaved roads that were made difficult by the unusually heavy rains, we made our final stop: the city of Aruanã, Goiás State. In that period of the year, we were the only outsiders in that city, which had been cut off by flooding and collapsed bridges. At that time (1978), Aruanã consisted of a single unpaved road, two or three restaurants where one could eat simple dishes, and a nearby Karajá village. Moreover, the town was embroiled in land conflicts. A conflict had also occurred between one landowner and one Karajá, which resulted in an uncomfortable atmosphere in the city.

After camping in the city (for less than a month), we received the opportunity to participate in everyday Aruanã life. Our camp, in the city, was located in the Araguaia riverbanks between the city limits and the unpaved road that led to the Karajá village. We often visited the village. We liked to visit it often, not only because we felt obliged to give special attention to the Karajá but also out of curiosity. We were always well received.

Then the river. The river affected me deeply. It made me dream and wonder how people lived along the river. A child of seven years, who was known as Zé, was always with us. He showed off his skills at climbing atop large stones and then plunging into the river. The brown, plentiful river was wide. The brownish, muddy river water contained many jaraqui (*Semaprochilodus* spp.) in addition to other fish. We had the opportunity to eat jaraqui after they were buried in the sand and cooked. The fish was cooked by making a hole in the sand with wood and fire, putting the fish above it, and then covering it with bananas leaves.

At night, local songs were performed by two “violeiros” (guitar men), who sang the local “moda de viola” (rural Brazilian song type). As a young woman born and raised at Copacabana Beach in Rio, these experiences were novel. It was the first time I heard “Chico Mineiro”, a “moda de viola” whose lyrics describe how two friends discovered that they were brothers only at the end of their lives, when one of the brothers found out Chico’s document after Chico has been shot to dead. A song lyric really representative of Brazil’s country side at that time.

### Human ecology

How people interact with the river and their food. The Araguaia was the scene of my first encounter with human ecology. My ideas and thoughts at that time concerned the relationships of the local people with the high fish diversity, in addition to how decision-making is embedded within the environment. During my time near the Araguaia river, I first entertained the idea of performing research on human ecology. The ideas I had at that time encouraged me to consider the relationships between the decision-making of the local people and the environment. Food is of interest in locations with high biodiversity, where extraction is an important aspect of the local people in context of their environment. During the trip to Araguaia, various interactions occurred while we flowed along the river, such as meeting locals upstream, riding in canoes with locals at night, and listening to stories about the local fish and the discoveries that Zé, the seven-year-old in the village, would tell us about daily; besides, other stories about the village, the fish and the river itself abounded. Ultimately, the feelings and experiences culminated during my second year of undergraduate research guided my decision to study human ecology.

## Motivating ideas and driving concepts

As a biology undergraduate, I was not yet familiar with the concepts of ‘emic’ and ‘etic’ [1, 2]. However, I realised that I was interested in what people were saying to me and how I could interpret (through other concepts and tools), what they were saying.

My time on the Araguaia was rich of nature and mind; this comprised my first step toward the pursuit of human ecology. The idea I had at that time played more instinctively rather than conceptually: I realised the distinctiveness of what I had in mind from a scientific perspective with respect to what people were telling me; I knew that biology was concerned, to a significant extent, with behaviour and that I was interested in understanding some aspects or mechanisms that underlined human behaviour; and I primarily wanted to understand the human ecology of food, particularly the relationship between people and their environment as mediated by food-related activities, such as extraction and consumption.

Later, I began to focus on emic and etic approaches, which were concepts developed by K. Pike in 1954 [1] and M. Harris [2, 3]. These concepts refer to insider (emic) and outsider viewpoints, such as that of the researcher (etic). These perspectives result in two distinct bodies of knowledge, the local and the scientific, as illustrated by researchers of local knowledge [4] and applied in my own research [5]. Later, as a complement to my research on the details of the interaction of humans and the environment, I added, more explicitly, ethnobiology to my research approach or agenda and investigated aspects of the perception, identification and the classification of the environment by local inhabitants [6].

In my research, I understand food as a phenomenon that embeds humans within an environment and that elucidates human decisions within their livelihoods and associated to their surroundings. This perspective resulted in studies on food taboos [7] and other decision processes that concern food choices and the food search, during which I also applied predictive models from ecology, such as the optimal foraging theory. The optimal foraging theory applies models from microeconomics to animal ecology to better understand human procurement, manipulation and consumption of locally available foods. A review of the optimal foraging models can be found in the classic ecological literature [8]. Initially concerned with animal ecology and then with humans, the optimal foraging theory was applied as a tool to understand the interaction of humans and food (for precursors of such studies, see [9]). The relationships of fishers with their decision-making processes, regarding which fish to pursue and consume, along with the commercial target species (the dilemma of ‘what to sell and what to eat’), could be analysed using the optimal foraging theory. These relationships and processes are discussed, for example, in studies on artisanal fishers of the Atlantic forest coast [10], and studies on the Negro River [11], among others. My interest in other ecological models and concepts developed from these studies, particularly the ideas related to food interaction and the extraction of resources, such as diversity indices [12] metapopulation [13] and resilience [14]. With respect to this last topic, associated with small-scale fisheries, I benefited from the work of F. Berkes, among others. However, I was also interested in the application and comparison of resilience concepts and ideas coming from evolutionary/ population ecology compared to studies on systems ecology [15, 16]. Additionally, cultural transmission models [17] became part of my interest as a means to understand behaviour, such as the behaviour associated with innovations and technological diffusion [18].

## Initial human ecology fieldwork: the Madeira and Negro Rivers (1981)

My academic research was strongly tied with theory and practice, collecting data through fieldwork, in the classical field ecologist methods (systematic samplings, for example) associated to anthropological methods (interviews, for example). My fieldwork was primarily conducted among artisanal coastal fishers, who have long used the sea as a food source and, by selling their catch in the local markets, they obtained cash for sustaining their livelihoods. Living far from the sea at Campinas in the state of São Paulo, my only compensation as a sea lover was to conduct research on the coast. However, I was struck by the parallels between the artisanal coastal fishers (known as Caiçaras) and the Amazon riverine fishers (known as Caboclos). Although they live in regions distant from one another, the Caiçaras and the Caboclos are rural people, who inhabit similar aquatic environments characterised by a high biodiversity of land and water resources. The comparison of these two groups has been crucial toward analysing the behaviour of people who extract resources from the forest, the sea, and the rivers. Analogous behaviours, such as food taboos, were found when the riverine Caboclos were compared with the coastal Caiçaras. For example, some of the local food taboos include carnivorous fishes, such as the surubim (*Pseudoplatystoma fasciatum)* in the Amazon and the tuna (bonito, *Auxis* sp., *Euthynnus alleteratus*) on the Atlantic Forest coast; these fish are forbidden, or not recommended for consumption by local people who are suffering from illness, by the riverine Caboclos and the coastal Caiçaras, respectively [7].

Additionally, something about the Amazonian rivers made me continually seek out opportunities for future fieldwork on them. I received various grants to pursue my research, most of which were from the São Paulo Agency (*Fundação de Amparo à Pesquisa do Estado de São Paulo* (*FAPESP*), which has supported my fieldwork throughout Brazil for my entire academic career (for both sea and rivers). Other agency, the CNPq, provided me with research productivity scholarships (and a PhD scholarship at UC Davis).

### Amazonian rivers

There is something in their smell and in the hot, perfumed air around them. This attraction is exuded in particular by one of the most beautiful rivers, the Negro River, in the Amazon. My experiences there have replenished my soul, rendering my fieldwork as meaningfully associated with *being in nature*, all of which allowed me to feel something ineffable and indescribable about the Amazon. One example of such feelings is what I would term the “nirvana” of riding in a canoe in the Negro River at night under a full or crescent moon while hearing the soft splash of water cut by the paddles and the canoe. In the tea-like water, in the black of night, the hot air was filled with smell of flowers. It was peculiar to be there as the sky loomed in the river water at the side of the canoe. The experience reminded me of a description of a typical tropical river, its natural surroundings and its people: a recall that I had by reading a book about Kerala, India, which the author [19] noted in detail the sensations, the colours, and the fragrance of the river and the interactions of the people and the environment in that part of India. Such special moments in the Amazon have captivated me throughout my life.

Fieldwork in Amazonia displays interesting juxtapositions. The riverine forest inhabitants and the townspeople share lively music and dancing. One experience illustrates such juxtaposition, which is emblematic of this tropical, vibrant culture. I was awakened in the middle of the night (3 AM) by loud local-style music with the Latin sounds and a similar Caribbean rhythm, typical of the area. This experience occurred in a ‘remote frontier’ near the border with Peru on the Breu River, which is a tributary of the Juruá River. The mixture of melancholy and calm from the river and the excitement of village life enhanced my capacity to experience life more vividly. My fieldwork on the Juruá was performed in 1993-1994. The work involved a challenging study of the riverine Caboclos in the first Extractive Reserve that was created in the Brazilian Amazon (the reserve was created in 1990). Extractive Reserves are protected areas that aim to conserve biodiversity and allow the permanent residence of local inhabitants within the reserve borders.

**Figure 1:**
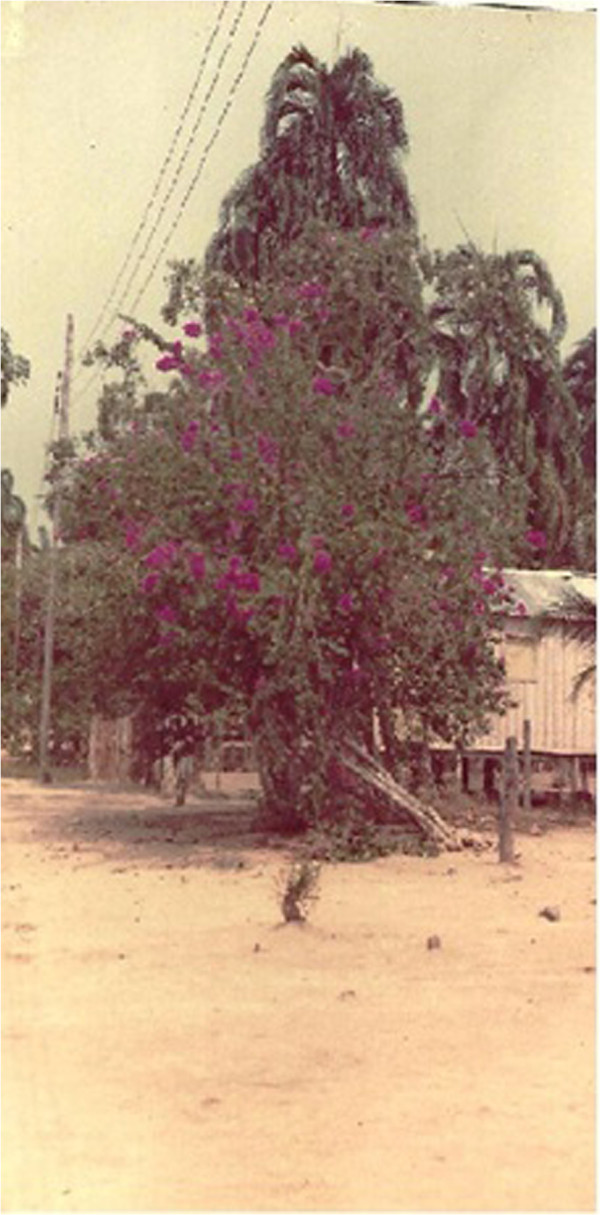
**A house in Vila Calama, Rondônia, Madeira river, Amazon, 1981.**

**Figure 2:**
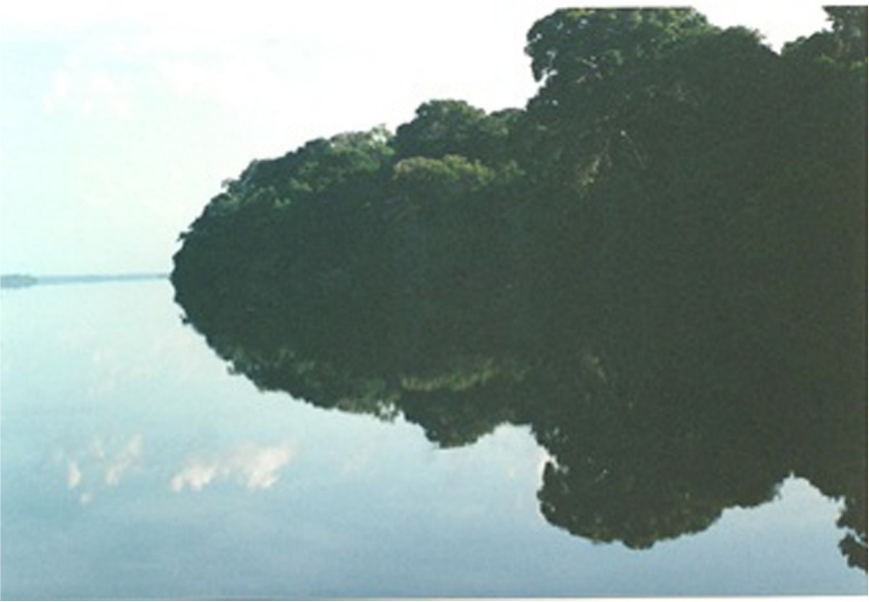
**The Negro river, Amazon, Brazil.**

When I finished my undergraduate (Biology-Ecology) at the *Federal University of Rio de Janeiro*, I entered to the Master degree in Ecology at the *Universidade Estadual de Campinas*, where I could not develop human ecology because of lack of supervisor in that field at that University (and in Brazil). Nevertheless, I was kindly invited to participate in a field trip led by a researcher from the INPA (*Instituto Nacional de Pesquisas da Amazônia*, Dr. Goulding), conducting interviews in Vila Calama (Madeira River) and on the Negro River (Figures 1-2). These results were presented at 1982 SBPC (*Sociedade Brasileira para o Progresso da Ciência)* meeting (Brazil). Those were my first outputs from a research in Human Ecology/Ethnobiology. Vila Calama (Madeira River) and Anavilhanas (Negro River) were then my first fieldwork projects, preliminary research, in both human ecology and ethnobiology, which encouraged me to continue research in that research area. I finally reached this objective at UC Davis, where I pursued a PhD (1985-1989), supported by CNPq, the Brazilian National Research Council (*Conselho Nacional de Desenvolvimento Científico*) and at UC Berkeley, where I attended courses in anthropology (Fall 1984). Later, when I was performing ethnobotanical research, I had the pleasure of visiting R. E. Schultes for a month at the Botanical Museum at Harvard, supported by a post-doc grant from *FAPESP* (São Paulo, Brazil).

## An island in the sea and extended fieldwork: Búzios Island, São Paulo, Brazil

After the first experience in the field in human ecology in the Amazon, I continue research in human ecology as a Ph.D. student in ecology at the University of California, Davis, in the United States. For my Ph.D. dissertation in this field, I investigated the artisanal fishers of Búzios Island, which is located 24 km off the coast of São Paulo State in Brazil.

My research at Búzios Island represents then my initial fieldwork in human ecology and ethnobiology, after the earlier preliminary experiences at Madeira and Negro Rivers in the Amazon. From 1986-1987, which was the period of my doctoral fieldwork, I was awarded grants to travel to the island for the fieldwork. My first grant was from the Alfred Sloan Foundation, which was obtained with the support of my dissertation advisor (major professor (Prof. Richerson)). Later, I received a grant through the CNPq, which was used to pay for transportation (in fishing boats).

The fieldwork consisted of 14 monthly visits. I collected data on fish catches, fish, fishing technology, and conducted interviews on food consumption, food preferences, plant extraction, and cultivation, among other topics. The journey to Búzios Island was always difficult, and in the winter, the rough seas made matters worse. I always depended on fishermen who were travelling to or from Búzios and sailed according to the weather and the need to bring in their catch (in those years, the fishers used styrofoam boxes with ice to preserve their fish). I often travelled from Campinas to São Paulo by bus and then to Ilhabela, where I could have a fisherman ferry me to Búzios. Often, I stayed overnight at Ilhabela and left for Búzios early in the morning (around 5 o’clock). The voyage required approximately two to three hours (by boat from Ilhabela to Búzios Island). When I stayed overnight at Ilhabela, two very kind persons that I got to know at Ilhabela during the research, my friends França and Winter, kindly hosted me in their homes.

Several trips to Búzios Island were made in the boats of the “Catarina,” who were fishermen from the state of Santa Catarina who fished near Búzios and on other sites of the São Paulo Coast. However, most trips were made in a small boat with a fisherman who had a family in Búzios but lived in Ilhabela. After several months, I arranged to have him pick me up at Búzios after finishing the monthly fieldwork. That arrangement was important. Without it, I could have been stuck at Búzios for several days.

### The fieldwork

Because I was influenced by the fascinating work of Malinoswski [20] before beginning my formal PhD programme, I was interested in the details of life in Búzios. Thus, I kept diaries, where I wrote about several aspects of the lives of the Buzianos, the people from Buzios Island. Búzios Island was difficult to reach and isolated at that time, and it was peculiar, interesting. A beautiful coast opened to me, every day from my window, at aristides and Dita’s home, the kind family that hosted me during fieldwork (no sandy beaches are found at Búzios Island). These diaries consist of detailed observations, which were organised by E. Camargo and published with me in 2006 [21]. The following paragraphs, which are partially excerpted from the book (21: pages 23-34), describe my first trip to Búzios Island.

#### First trip to Búzios Island for human ecology research: September 1986

(Shortened version translated and adapted from Camargo and Begossi, 2006).

*There is no doubt that this first trip was the most outstanding and memorable one, particularly for someone who was born looking at the sea in Copacabana, Rio de Janeiro. The sensation of viewing of the huge and vast sea as I crossed from Ilhabela to Búzios Island was fulfilling. On that day, I was certain that I had chosen the right profession. Fields like these would be my place of work, my office, my unpolluted scenario, lightly touched landscape. I was sure I did not want to be at home at night, arriving from the work and sitting in an armchair, taking off my high heels, and relating the same daily routine to my listener. I wanted to see the world with Icarus’s eyes.*

*After I left Campinas and headed toward my first days of fieldwork, everything became a part of my fieldwork diaries. The fishers’ names, the names of local institutions, the costs of the trips, and the small and informal talks.*

*From Rio de Janeiro, the trip turned south on the Rio-Santos highway and with a side view of the Atlantic Forest coast. From Campinas, the bus travelled the Tamoios highway to São Sebastião. There, I took a ferryboat to Ilhabela, where I was transported by small boat or motor canoe to Búzios Island.*

*During those trips in small boats, I sat near the prow far from the smell of the diesel smoke coming from the motor, which can easily nauseate a passenger. It was early in the morning, and the sea was glossy, slippery, and very calm. The sea resembled a lagoon, but only the afternoon before, the windy day had castigated the sea.*

*When you leave the Ilhabela harbour, you head north and admire various beaches and islands, including Mercedes, Siriúba, Arrozal, Ponta Azeda, and the northeastern point of Ilhabela Island, known as Ponta das Canas. From there, the fisherman turns to follow the course to Búzios Island. Appearing on the right is Sumítica Island, and on the left is Vitória Island.*

*The grey patch in front slowly became the approaching Búzios Island, with its blue-grey stones. On one side, at an elevation of 413 meters, are forests. On the other side, a deforested space is allocated for manioc cultivation. Wood was obtained above in the forest to construct houses and canoes. The coordinates for Porto do Meio, which is the island’s main harbour, with 24 houses, are 23*^*o*^*47’63”S latitude and 45*^*o*^*08’92”W longitude (GPS Magellan, 1998). Only a few meters before reaching Búzios Island, the houses of Porto do Meio became visible, with houses in the highlands and the rocky coast showing its stones close to the harbour.*

**Figure 3:**
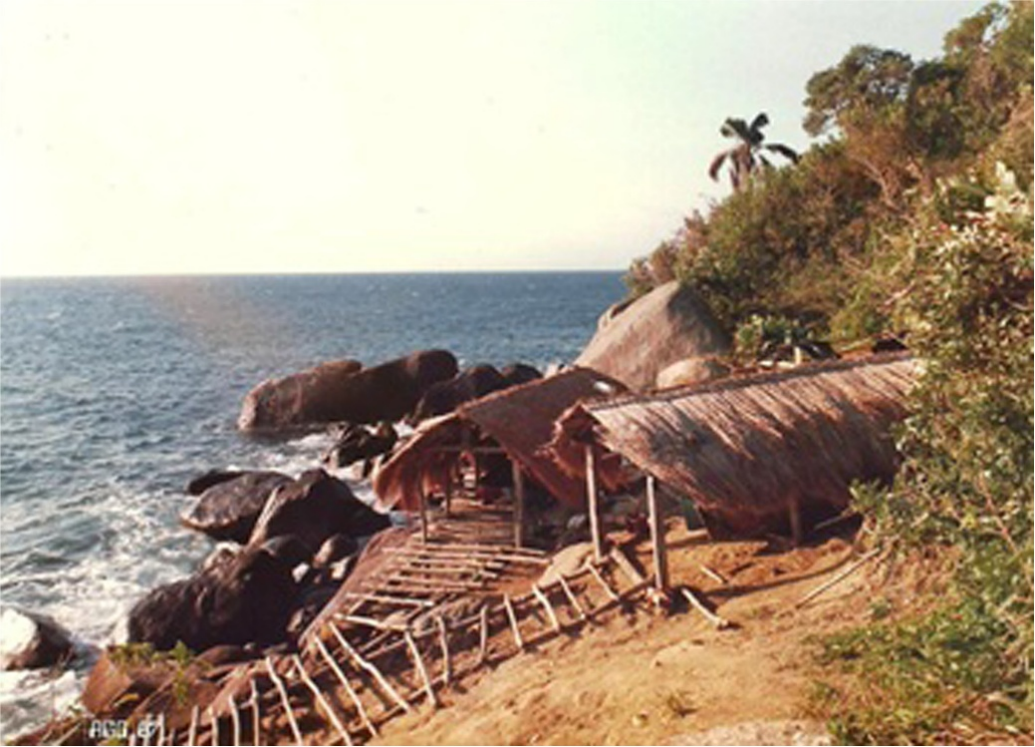
**A harbour at Búzios Island, São Paulo State, SE coast of Brazil.**

*Six harbours represent the familiar nucleus of Búzios Island: Porto do Meio, Guanxuma, Jerobá, Pitangueiras, Mãe Joana, and Costeira* Figure 3*. At Porto do Meio harbour, there were simple fish depots, where the fish were weighed by the buyers and stored in styrofoam boxes with ice. The local scales were made of brass trays. The weights were made of stones and calibrated in one of the nearby cities, either Ilhabela or São Sebastião. Such weights served the fishermen and buyers despite the constant conflicts that resulted from the suspicions of the fishermen regarding the weight stated to them by buyers.*

*I arrived close to noon. I was received by several inhabitants who went to the harbour to help me escalate the harbour, which was constructed of rounded logs and stones near the houses. One old fisherman, Argemiro, was particularly helpful, and he became a key informant during the remainder of my fieldwork.*

*I was hosted by a couple with a daughter. They hosted me for nearly the entire period of the fieldwork, which consisted of monthly visits over 14 months. I provided the provisions, and I was glad to take their orders for food that they enjoyed. Aristides and Ditinha were always kind and helpful.*

*I was quartered in a room in a wooden house from which I could observe the sea and the movements of the fishers with their hooks as they searched for squid or when they returned from visiting their nets. It was interesting to observe the fishers. I often awoke at 5 o’clock in the morning and could occasionally observe a fisher visiting a net close to his home. I could then run to the harbour to collect data on the fishing trips (a systematic monthly sampling of fishing trips was one of the methods used during the fieldwork). To avoid insects, I followed the Amazonian practice of sleeping in a hammock, which I usually left at Búzios between trips for the local children to enjoy.*

*At that time, most houses on Búzios Island were constructed of local wood. Most were simple but clean, including the one in which I was hosted. There was only one bathroom close to our house, which served four or five houses, and it consisted of a ground space that was hidden by rocks and a bed sheet that was used as a cover (as a door) for privacy.*

*Water from the top of the mountain was distributed through hoses to the faucets in the backyards of the houses, where dishes were washed and showers taken.*

*The purpose of this first trip to Búzios Island was to become acquainted with the people and to plan the fieldwork. I went to all of the houses to explain the research project. I understood from the first trip that Búzios’s population consisted of three main families, all of which were related. In those initial talks, I could conclude that the Búzios Island residents were related to families from nearby Vitória Island and that some of the inhabitants of Búzios came from Portugal, Ilha Grande, São Sebastião and Rio de Janeiro.*

*As I began the fieldwork, I benefited from the research presented in books by Emilio Willems in collaboration with Gioconda Mussolini (1947) and by Euclides da Cunha (1902) (Ilha dos Búzios, Anais do Nono Congresso de Geografia, vol. V, Rio de Janeiro, Brasil, 1944). A copy of Willem’s book was given to me by a friend, M. Campos, who visited Búzios and had done some fieldwork on ethnoastronomy at that time at Búzios; he was the one who suggested me to do my Doctoral fieldwork in that island.*

*On my second day on Búzios Island, I visited the garden where vegetables were planted, located close to the houses. The manioc garden was located a little farther from the houses than the vegetable garden.*

*Meals were eaten with the host family and included fish, rice, beans, and some green vegetables. The meals were tasty, particularly when fried squid was served. The best squid I have ever tasted was prepared on Búzios Island by Ditinha. In the winter, when fish were scarce, the children used to eat birds served with beans. Far from the coast, amberjacks (Seriola* spp*.) were caught at Búzios, which were tasty.*

*In the afternoon, after the fish was landed and were brought in, I had the opportunity to listen to stories and hear some of the local poetry. At first, the local people were shy, but then they quickly began to tell me the details of their lives. From that point onward, I knew that I was interested in all matters related to fish, nets, food, local dishes or recipes, religion, plants, and taboos. That first week, I began understanding the nets (measuring the nets and understanding the target fish, among others).*

## Intellectual challenges

Nature exerts a variety of stimuli. However, deep inside, the brain operates by making innumerable associations, suggesting theories that are confronted with observations. What I refer to here as “deep inside” are the challenging questions that inspire us to perform research. Often, these questions are associated with one’s reading background and experiences we carry within. I am certain that my reading influenced my career in terms of the questions I have had regarding human-nature interactions and the tools I have chosen to answer those questions. In chronological order, I summarise the readings that have had a special influence on my life and research.

- *Home:* I came from parents that were always excited reading and discussing points of views. My mother from a left side, and father from a right side. My mother as having ‘freedom’ as the core of her life; my father trying to convince her about ‘order’. But, the important point is that books, especially on philosophy and history were in high esteem at home and subject of discussions at lunch or at supper. Plato, Kant, Spinoza, Schopenhauer, as well as many others, were in the core of much talking, among others. Philosophy enriches the mind, and naturally, enriches life and research.
- *Student movement:* During my undergraduate years in Rio at the Federal University, I read Hegel, Marx, Engels, Lenin, and poets such as Brecht, among others. I was part of the movement against the military-totalitarian government that ruled Brazil for approximately 20 years and a member of student and political organisations. Those were intellectually rich years during which I read widely a high diversity of themes from philosophy to political economy. Through the political organizations I was a member, I had the opportunity to enter actively the student movement experiencing writing, theatre and, of course, parades and rallies. Excited talks enriched those years; also at home, especially with my mother who also participated in the student movement in the forties, when she was at the Universidade do Brasil.
- *Readings during undergraduate and graduate education in evolution:* Under the guidance of a professor (Prof. Iglesias) during my undergraduate programme, we read and discussed on evolution: Darwin, R. Lewontin, E. Mayr and G. Simpson, among others. Following those years, I continued reading on evolution during my Master’s programme at Unicamp, influenced by my advisor’s interests in evolutionary ecology (Prof. Benson), a field that continued to interest me. As a graduate student at UC Davis, a wide horizon was opened under the influence of P. J. Richerson, my supervisor in ecology and human ecology (which were the subjects of my PhD studies) who had been studying, modelling and publishing on cultural transmission models [17]. At that time, I was also favourably impressed by the literature on cultural anthropology, which I read with great pleasure during a course at UC Davis (Prof. Orlove). This literature included texts by J. Steward, M. Harris, and other precursors of cultural ecology, as well as studies on small-scale fisheries by authors such as J. Acheson, J. Cordell, B. McCay, K. Ruddle, B. Johannes, and the classic *Those who live from the sea,* by M. E. Smith, which is pleasantly detailed. Certain aspects of economic anthropology also interested me, for example, the work of M. Bloch and E. Terray, among others. Other influential reading included work by sociobiologists, such as R. Dawkins, R. Alexander, R. Trivers, and of course, E.O. Wilson; also readings by researchers who conceived of genes and culture as coevolutionary processes, and who contributing on theory and modelling on cultural evolutionary processes [17, 22, 23], among others).

Then, the next phase of my readings in my career focused on Human ecology. Ethnobiology, and Fisheries, among others. Human ecology had already become part of my research life and I benefited from the inspiring meetings of the Society for Human Ecology (SHE). But, Ethnobiology as a branch of it, or complementary to it, slowly gained more of my attention, particularly because the field embedded questions (besides others on people and natural resources), related to the functions of the mind, such as perception and the categorisation of nature. Here, authors such as C. Brown and E. Hunn and linguists such as N. Chomsky were of interest, in addition to B. Berlin, among others. In the following years, my interests dynamically snowballed, so to speak, following the influences of research questions, new interests and local circumstances. Food acquisition (Ethnoecology), Ethnobotany and Ethnomedicine were areas of significant interest in the first 10 years of my career after I completed my PhD. In particular, in the beginning, a botanist collaborator, H.F. Leitão-Filho, stimulated me to enter the field of ethnobotany. Unfortunately, I had to forgo Ethnobotany and Ethnomedicine as research areas in Brazil because of the substantial restrictions and difficulties imposed by Brazilian laws [24] regarding fieldwork in these research areas in Brazil.

Afterward, my interests diversified as new and intriguing questions continued to appeal to me. However, I focused particularly on certain topics, which I summarise as follows: the perception of nature; the use of nature; the decision-making processes when extracting and using natural resources; the use of aquatic space; and the means (research applications) to conserve the culture and the biodiversity of small-scale fisheries (management and co-management). Then, Ethnotaxonomy, Ethnoecology, and Ethnomedicine, with sub-interests in Ethnobotany and Ethnoichthyology, became the primary focus of my studies. In recent years, co-management with a focus on small-scale fisheries (and associated local and scientific knowledge), including ecological economics, have become my chief areas of interest. For a long time, ecological economics has navigated in my interests because there are a substantial number of models used in ecology that are derived from economics and microeconomics (for example, the optimal foraging theory). However, the ecological economics of the management of Brazilian small-scale fisheries is a study area I have entered when collaborating as post-doctoral researcher, supported by CNPq, in Rio, with P. May. Generally, my research included decision-making processes and variables regarding food procurement and food choices; food taboos; classification of nature (folk taxonomy); fishery management;

In particular, Ethnoecology was the core of the fishery studies. Collaborative research with fishers sum up efforts culminating with the foundation of the *Fisheries and Food Institute* (http://www.fisheriesandfood.org) in 2006.

## Fieldwork methods

**Figure 4:**
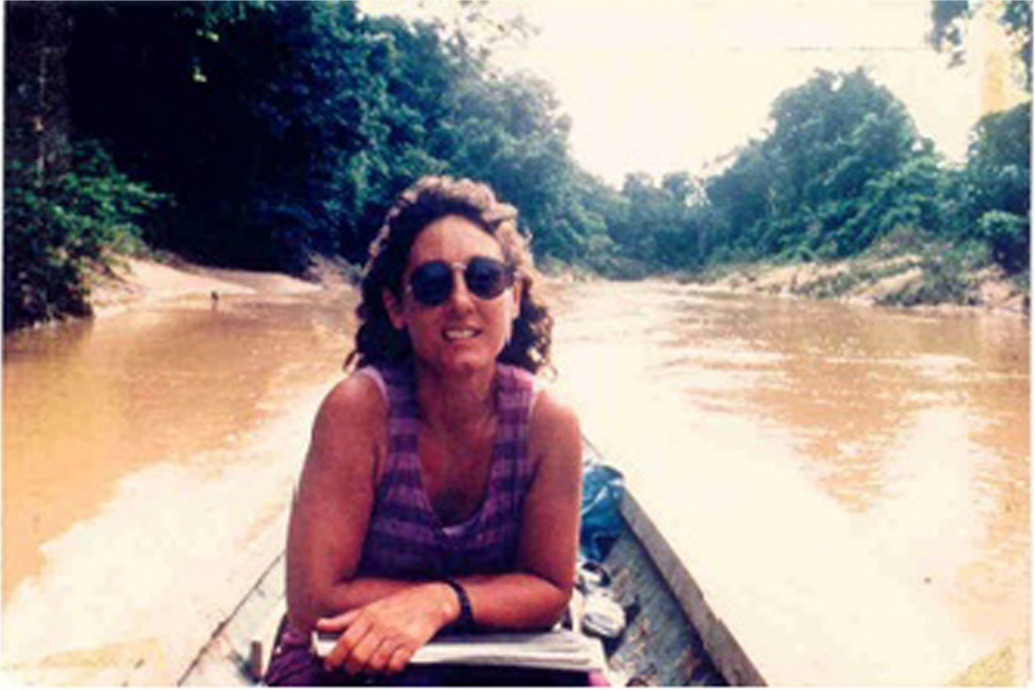
**Research with riverine caboclos (fishers) by sampling houses by boat in the Juruá river and tributaires, Acre, Amazon.**

As ethnobiologist and human ecologist, nature and fieldwork is a fundamental research side. There are two primary procedures that I (and our team) have used and adapted to study the sea and the rivers. I refer to them as systematic sampling and interviews. Systematic sampling enables me to answer questions regarding, for example, fish production and food consumption, to discriminate the etic aspect of research. Interviews enable me to assess and thoroughly address the cognitive, decision-making processes, as well as the emic aspects of research and Ethnobiology.For rivers, one method used to conduct interviews is to stop to visit the homes of individuals who live on the riverbanks by canoe following a transect of all of the homes, or half of them (every other home), so that a percentage, taken at random, of homes reached and visited is possible (Figure 4). For the sea, interviews are conducted in the villages, through samples or simply by conducting interviews in all the village homes (Figure 4).

The mapping of fishing spots: for more than 20 years, I have travelled the coast of Brazil and the rivers of the Amazon by boat and canoe. On the coast, the fishing spots are marked from the boats with the help of fishermen and by using GPS. Another method to mark the fishing spots is the so-called fishing approach, in which a fishing boat is approached during fieldwork and information is gathered from the fishermen while they fish, including the fishing spot’s coordinates. A book with maps and information was published in 2013 [25].

**Figure 5:**
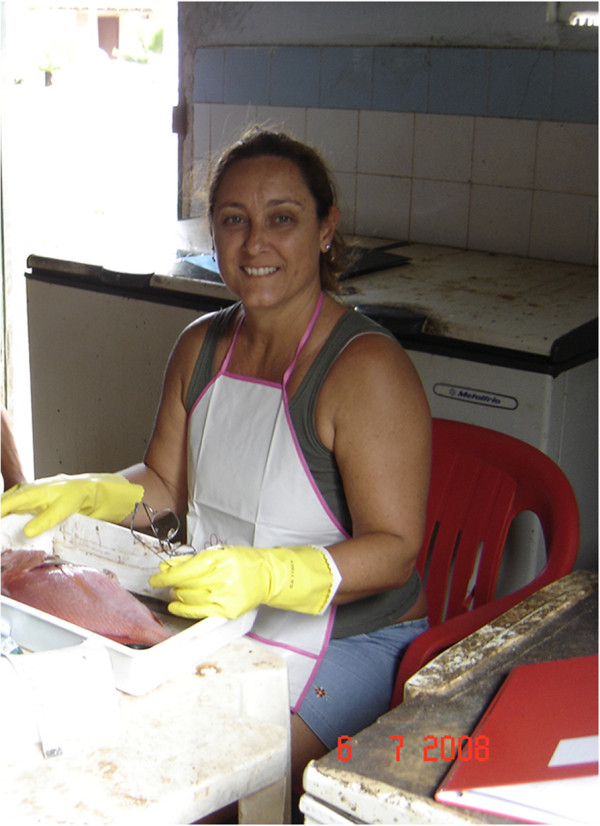
**Sampling stomachs and gonads of snappers at Porto do Sauípe, Bahia State, Brazil, in a local fish market.** Fishermen were helpful and liked to observe and comment upon the work, besides kindly arranging the table for me.

Sampling fishing trips: in particular, systematic sampling is performed to collect data on fishing trips. A protocol is followed that includes questions on fish species and the fishing spots visited. Moreover, food consumption recall is used to gather data on the food items consumed during meals.Fish biology: to collect data on the diet and the reproduction of fish, I work closely with the fishermen at landing points or in the local fish market (Figure 5). The fishermen have been usually kind and helpful.

## Concluding remarks

My work at Búzios Island was one of the most in-depth fieldworks I have ever performed. I entered into the fieldwork with great curiosity and an open mind. Using the data collected at Búzios Island (and other sites), I published several papers, and I still have additional data to publish. Earlier research on Búzios Island is worth to be cited here [26, 27]. Maintaining an open mind in the field enabled me include subjects that I had not initially planned to investigate. I became open to these long-lasting research interests, some of which I continue to examine when collecting data and writing papers. Other villages in Brazil were visited for research among riverine fishermen from the Amazon (in special the Araguaia, Juruá, Negro and Tocantins rivers) and among coastal small-scale fisheries, from Santa Catarina State up to Ceará (the NE coast). The research interests include the following:

- Territoriality. This topic involves the spots used by the fishers and the informal division of net-fishing grounds among families [28]). This research helped me understand the use of aquatic space, which is important in the management of artisanal fisheries [25].
- Local knowledge (and Ethnobiology). The knowledge that individuals possess regarding natural resources, particularly local knowledge about plants and fish extracted for food, medicine, among others, as well as local knowledge on fish biology and ecology, along with and Ethnotaxonomy.
- Food taboos and medicinal animals. Knowledge of these topics helps explain food aversions and the mechanisms of related behaviours. Here, emic and etic approaches are enlightening. The local experience and knowledge interacting with scientific knowledge and their interaction are examples.

Fieldwork is an essential part of biology (and of cultural anthropology). I have the impression that there is something in addition to a research interest that motivates individuals to choose disciplines that involve fieldwork and interest in Ethnobiology and Human Ecology. This “something” is the possibility to enter the minds of others, to enter new cultures, and to appreciate the diversity of life in its natural and cultural aspects.

Since Búzios Island, I have performed research at other locations in Brazil in the coastal areas of Pântano do Sul (Florianópolis, Santa Catarina State, southern Brazil), through Itacimirim and Porto do Sauípe (Bahia State), Riacho Doce (Maceió, Alagoas State), Ponta Negra (Natal, Rio Grande do Norte State), Mucuripe (Fortaleza, Ceará State, northeastern Brazil) and on the rivers Araguaia (a returning to my beginnings), Tocantins, Juruá, and Negro, among others. Extractive Reserves, as well as Sustainable Development Reserves in the Amazon (as is Mamirauá) I also visited for fieldwork.
